# Effect of rice variety and blending proportion on the proximate compositions, minerals and phytic acid contents of bread from rice-teff blend

**DOI:** 10.12688/f1000research.6201.1

**Published:** 2015-05-13

**Authors:** Sintayehu Legesse, Solomon Worku, Geremew Bultosa

**Affiliations:** 1 Department of Agricultural Engineering, Adama Science and Technology University, Adama, Ethiopia; 2 Department of Food Science and Postharvest Technology, Haramaya University, Dire Dawa, Ethiopia; 3 Department of Consumer Sciences, University of Swaziland, Swaziland; 4 Department of Food Science and Technology, Botswana College of Agriculture, Gaborone, Botswana

**Keywords:** Blending proportions, Gluten-free bread, Response surface methodology

## Abstract

Development of bakery products containing rice ( *Oryza sativa, Linn*.) and teff ( *Eragrostis tef*) could have potential health benefits due to their gluten free nature. Nine experimental runs were generated using custom design by JMP 8 software. The effect of two factors, rice variety (Edeget, X-jigna and Nerica-4) and blending proportions of rice and teff (0.5:0.5, 0.7:0.3 and 0.9:0.1) were studied. The data analysis was conducted using SAS software package for the mean comparison and custom design by JMP 8 software. Response surface methodology was applied to study the interaction effect of the main factors and to generate the predictive equations. An optimal value (1.60%) of fiber was obtained when the proportion of the blend was 50% Edeget and 50% teff because teff grain is high in fiber. A maximum value (10.75%) of protein was obtained when the proportion of the blend was 70% Nerica-4 and 30% teff. Carbohydrate was optimal (81.37%) when 90% Edeget and 10% teff were blended because rice grain is high in carbohydrate. Optimal iron content (12.97 mg/100g) was obtained when the proportion of the blend was 50% Nerica-4 and 50% teff because teff grain is high in iron. Optimal zinc content (4.14 mg/100g) was obtained when the proportion of the blend was 50% X-jigna and 50% teff. The optimal value (61.25 mg/100g) of calcium was obtained when the proportion of the blend was 50% Edeget and 50% teff.  Optimum (lower) value (0.31mg/g) of phytic acid was obtained when the proportion of the blend was 90% Nerica-4 and 10% teff because rice grain is lower in phytic acid content. It was concluded that rice variety and rice-teff blending proportion had a significant effect on the physico-chemical properties of rice-teff blend bread. An optimal nutrient blend (high in nutrients, low in anti-nutrients) was obtained when 70% Edeget rice variety was blended with 30% teff. All the derived mathematical models for the various responses were found to fit significantly to the predicted data.

## Introduction

Rice ( *Oryza sativa, Linn*.) is the most important cereal in terms of the numbers of people it nourishes. Traditionally, it has been the staple food and main source of income for hundreds of millions of people throughout the world. It holds the 2 ^nd^ place next to wheat in its importance as a food cereal in the human diet, and produces more food energy than other cereal grains ( Aklilu *et al.,* 2002). Rice can play a very important role in human nutrition especially for developing countries like Ethiopia because it is a popular, gluten free source of carbohydrates, B-vitamins and is non-allergenic to celiac patients ( Dziezak, 1991). It also contains about 6–7% of high quality protein ( Choudhury & Gautam, 1998).

In Ethiopia, rice is consumed in different forms; as a substitute for other major cereals mainly for injera (by mixing with millet and teff, *Eragrostisteff*), in bread (alone), as cooked rice, in brewing local drinks (“Farssoo” and “Araqee”), porridge and Kinche (splatted and cooked oats). Thus it fits well into the food habits of Ethiopians. Moreover, rice is a good source of income for farmers and has a higher yield and price than that of teff in the local market. However, it is claimed that consuming rice brings constipation especially in children due to its low fiber content. It is also poor in mineral and fat content. Mixing rice with other cereal crops can improve these problems ( Aklilu *et al.,* 2002).

On the other hand, rice flour has become an attractive ingredient in the processing industry due to its unique attributes such as white color, hypoallergenicity and ease of digestion ( Kadan *et al.,* 2003). It has also an excellent expansion property because of its high starch content and is well suited to thermal processing to produce a variety of food products ( Ibanoglu *et al.,* 2006).

Teff is a staple cereal crop indigenous to Ethiopia that supplies a large proportion of the daily calorie intake for the majority of the Ethiopian population ( Seyfu, 1997; Bultosa & Taylor, 2004). According to a Central Statistical Authority (CSA) (2010/11) report, teff cultivation takes up the largest amount of land under cereal cultivation (27.49%, 2.72 million hectares) and is the third largest crop (after maize and wheat) in terms of grain production (19.92%, 34.34 million quintals) in Ethiopia. As it is in high demand and it has a high market value, farmers earn more from growing teff than growing other staple crops. At present, teff is produced predominantly by smallholders who rely on a rainfall. Teff cultivation as a cereal food grain is restricted to Ethiopia, except in very small quantities in Eritrea and recently, in Israel, the Netherlands and U.S.A. Teff is also gaining popularity as health food ( Spaenij-Dekking *et al.,* 2005).

Teff is as nutritious as major cereals like barley, oats, rice and wheat and even better in some aspects ( Seyfu, 1993). It is a rich source of B-vitamins and minerals and is considered to be an excellent source of essential amino acids with higher levels than wheat and barley ( Seyfu, 1993). The grain is small, with an average length and width of 1.00 to 1.20 and 0.59 to 0.75 mm, respectively ( Bultosa & Taylor, 2004; Zewdu & Solomon, 2007). This makes it inconvenient to separate the germ from the bran and so the germ and the entire seed are consumed. This results in better nutrient provision and higher fiber content.

Studies made on the utilization of teff are few and have been limited to the biological and biochemical changes taking place during the fermentation process ( Asrat & Frew, 2001). However recently there has been a growing interest to develop new products from teff using modern processing techniques, like extrusion cooking to harness its potential ( Laike, 2006). Research into these techniques has been limited in Ethiopia.

Due to the many functional as well as nutritional properties of rice, it can be used for gluten free bread-making in combination with teff, which serves to compensate its limitations. Teff is gluten free in nature and has the potential to increase fiber, fat, B-vitamins and minerals consumption in the products. This paper seeks to characterize rice-teff bread by reporting its proximate compositions, including minerals and phytic acid content.

## Materials and methods

### Experimental materials and bread sample processing

Three varieties of rice ( *Oryza sativa,* Linn.) and teff ( *Eragrostis teff*) grain were obtained from Adet Agricultural Research Center (AARC) and Debre Zeit Agricultural Research Center (DZARC), Ethiopia respectively. The rice was manually cleaned, milled so as to be able to pass through 710 µm sieves ( Laike, 2006) and blended with the required ratios (defined below).

Bread was baked using straight-dough method as described in the AACC (2000) method № 10 - 10B. Fresh baked bread was dried for 24h at 65°C in an oven (Model: 101-1A; Tianjin Taisite Instrument Co., Ltd, Tianjin, China) and ground by mortar and pestle to pass through a 750 µm sieve. This sample was kept in sealed plastic bag at refrigeration temperature (5°C) and was used for proximate composition, minerals and phytic acid analysis.

### Experimental design

The effect of rice variety and blending proportions of rice and teff on bread composition were studied using a custom design. The proportions of rice to teff ranged from 50 to 90% whereas teff from 10 to 50%. Rice bread (100% rice) was used as a control. Each formulation had nine runs and was done in triplicate.

In building the model, a regression equation was established to describe the relationship between the response Y and variable X. A second order model was generated for the two mixture components as follows:

Y = β _1_X _1_ + β _2_X _2_ + β _12_X _1_X _2_ + β _3_X _3_ + β _4_X _4_ + β _5_X _5_            (1)

Where: Y is the predicted response; β _1_ and β _2_ are linear coefficients; β _12_ is the interaction coefficient; β _3_, β _4_ and β _5_ are varietal coefficients and X _1_, X _2_, X _3_, X _4_ and X _5_ are independent variables.

### Determination of ash content

Total ash was determined according to AOAC (1995). Approximately 3g dried bread sample was carbonized on hot plate and transferred to a muffle furnace (MF 120, Ankara TURKEY) and combusted at 550°C until ashing was completed (over 12 hrs). The residue was cooled to ambient temperature in desiccators (Nalgene Model 5317-0120) and then, the total ash was calculated.

### Determination of crude fiber contents

The crude fiber was analyzed according to AOAC (1995). Approximately 3g dried bread sample was digested with 1.25% sulfuric acid and washed with distilled water and further digested with 1.25% sodium hydroxide, filtered through coarse porosity (75μm) crucible in apparatus at a vacuum of about 25 mm. The residue left after refluxing was washed again with 1.25% sulfuric acid at near boiling point. The residue was dried at 100°C for 2 hrs, cooled in a desiccator. After being dried the sample was ashed at 550°C for 2 hrs; after ashing the sample was cooled in a desiccator. Total crude fiber was then calculated.

### Determination of crude fat contents

The crude fat analysis was determined by Soxhlet extraction method in accordance with AOAC (1995), method 920 - 85. A thimble with approximately 2g dried bread sample was placed in a 50 ml beaker and dried in an oven for 2 hrs at 110°C. The sample contained in the thimble was extracted with petroleum ether in a Soxhlet extraction apparatus for 8 hrs. After the extraction was completed, the extracted fat was placed in a fume hood to evaporate the solvent on a steam bath until no odor of the solvent was detectable. The extracted fat was then dried in an oven for 30 minutes at 100°C. Finally, it was removed and cooled in a desiccator. Crude fat content was calculated.

### Determination of protein contents

The total nitrogen content of the sample was analyzed by micro-Kjeldahl method as described in AACC (2000) Method № 46 – 11. Approximately 0.3g dried bread sample was digested in a flask containing 2.5 mL of a mixture of H _2_SO _4_ + Se (100 mL) and salicylic acid (7.2g) and three pieces of boiling chips. The content of the flask was digested at a temperature of 350°C on the digestion apparatus until the digestion was completed (the digest becomes clear). The acid digest was allowed to cool at room temperature. The digested sample was transferred to a distillation unit (Model UDK-142, Europe) and distillation was under taken by adding 30 mL of distilled water followed by 25 mL of 40% NaOH and connecting it to distillation apparatus whose outlet tube was immersed in 25 mL of 4% boric acid solution. The distillate (about 150 mL) was collected and titrated by standard acid (0.1N HCl). Urea was used as a control in the analysis.

### Determination of iron, zinc and calcium contents

These were determined using an atomic absorption spectrophotometer (Model: 210 VGP spectrophotometer, Buck Scientific, East Norwalk, CT, USA) after digestion of approximately 3.0g dried bread using air-acetylene as a source of energy for atomization ( AACC, 2000). For iron content determination absorbance was measured at 248.3nm and iron was estimated from a standard calibration curve (3–8µg Fe/mL) prepared from analytical grade iron wire. For zinc content determination, absorbance was measured at 213.8nm and zinc level was estimated from a standard calibration curve (0.1–1.0µg Zn/mL) prepared from ZnO. For calcium content determination, absorbance was measured at 422.7nm after addition of 1% lanthanum (i.e., 1mL La solution/5mL) to sample and standard to suppress interferences. Calcium content was then estimated from standard solution (0.1–1.0 µg Ca/mL) prepared from CaCO _3_.

### Determination of phytic acid

Phytic acid was determined after 0.25 g of flour sample was extracted with 12.5 mL of 3% Trichloroacetic acid (TCA), precipitation of phytate as ferric phytate with addition of 4 mL of FeCl _3_ (2mg/mL) ( Poiana *et al.,* 2009) followed by phytate phosphorus (Ph-P) analysis ( Morrison, 1964) using a conversion factor i.e., phytate = P × 3.55 ( Poiana *et al.*, 2009).

### Data analysis

At least triplicate data were analyzed by ANOVA and modeled using the statistical software JMP ^TM^ 8, 2008 (by SAS Institute Inc., Cary, NC, USA). Response surface methodology was applied to the experimental data using JMP version 8 to study the interaction effect of the main factors and to generate the prediction equations. Mean comparison has been done by Duncan’s Multiple Range Test (DMRT) by SAS 9.1.3. Mean values were considered at 95% significance level.

Data for JMP analysis The displayed data was used to study the effect of rice variety, proportions of rice and tef, the interaction effect of the main factors on bread quality and to generate the prediction equations using statistical software JMP 8, 2008 (by SAS Institute Inc., Cary, NC, USA) ( Legesse *et al.*, 2015a). Click here for additional data file. Copyright: © 2015 Legesse S et al. 2015 Data associated with the article are available under the terms of the Creative Commons Zero "No rights reserved" data waiver (CC0 1.0 Public domain dedication).

Data for SAS analysis Data used for the mean comparison using Duncan’s Multiple Range Test (DMRT) by SAS 9.1.3. Mean values were considered at 95% significance level. Where: N is Nerica-4 rice variety, X is X-jigna rice variety and E is Edeget rice variety ( Legesse *et al.*, 2015b). Click here for additional data file. Copyright: © 2015 Legesse S et al. 2015 Data associated with the article are available under the terms of the Creative Commons Zero "No rights reserved" data waiver (CC0 1.0 Public domain dedication).

## Results and discussion

### The effect of rice variety and blending proportion on proximate composition of bread

The ash content of the product ranged from 2.71–3.74% ( Table 1). The ash content of the control was 2.7%, which was significantly (P<0.05) increased on blending with teff at different proportions. This is mainly due to the higher ash content of teff flour as compared to rice flour. Bultosa (2007) reported that teff grain ash content ranged from 1.99 to 3.16% with mean of 2.45%. This is due to teff grain’s proportionally high bran content ( Bultosa & Taylor, 2004). Ash content indicates milling performance by indirectly revealing the amounts of bran removed. The highest ash content (3.74%) was obtained when 50% X-jigna rice variety was blended with 50% teff and lowest ash content was obtained when 90% Edeget rice variety and 10% teff were blended.

**Table 1.** The effect of rice variety and blending proportion on the bread proximate compositions.

Runs	Ingredients		V	Ash (%)	Crude fiber (%)	Crude fat (%)	Crude protein (%)	Carbohydrate (%)
	Rice	Teff						
1	0.5	0.5	E	3.48±0.03 ^c^	1.60±0.00 ^a^	1.90±0.05 ^a^	9.74±0.14 ^a^	77.84±0.09 ^f^
2	0.7	0.3	E	3.10±0.04 ^e^	1.40±0.05 ^b^	1.12±0.01 ^e^	10.30±0.01 ^a^	79.66±0.07 ^cd^
3	0.9	0.1	E	2.71±0.00 ^g^	0.63±0.04 ^f^	0.85±0.03 ^f^	9.71±0.00 ^b^	81.37±0.07 ^ab^
4	0.5	0.5	X	3.74±0.01 ^a^	1.35±0.04 ^b^	1.81±0.03 ^b^	10.38±0.45 ^a^	77.95±0.59 ^f^
5	0.7	0.3	X	3.12±0.02 ^e^	1.24±0.04 ^c^	1.19±0.01 ^e^	10.52±0.15 ^a^	79.14±0.16 ^e^
6	0.9	0.1	X	2.87±0.02 ^f^	1.00±0.07 ^d^	0.90±0.06 ^f^	10.49±0.16 ^a^	79.95±0.30 ^c^
7	0.5	0.5	N	3.55±0.01 ^b^	1.56±0.05 ^a^	1.65±0.02 ^c^	10.26±0.01 ^a^	79.20±0.05 ^ed^
8	0.7	0.3	N	3.22±0.02 ^d^	1.53±0.09 ^a^	1.29±0.09 ^d^	10.75±0.19 ^a^	79.07±0.07 ^e^
9	0.9	0.1	N	2.86±0.01 ^f^	0.80±0.01 ^e^	0.87±0.06 ^f^	10.58±0.59 ^a^	81.09±0.49 ^b^
Mean				3.14±0.35	1.16±0.40	1.22±0.42	10.33±0.40	79.70±1.33
Range				2.71–3.74	0.63–1.60	0.85–1.90	9.71–10.75	77.84–81.37
Control	1	0	X	2.70±0.05 ^g^	0.46±0.01 ^g^	0.63±0.01 ^g^	9.74±0.14 ^b^	81.78±0.03 ^a^

Values are in means ± standard deviation on dry matter basis. Means within a column with the same letter are not significantly different at 95% probability levels. Where: V=rice variety, E=Edeget, X=X-jigna and N=Nerica-4.

The combined effects of rice variety and blending proportion on ash content was significant (P<0.0001). All the linear terms, interaction term (R*T) and Edeget and X-jigna rice variety were significant for ash content ( Appendix table 1.4). The following model ( Equation 2) was developed to predict the crude ash content of the rice-teff blend bread.

AC = 2.69R + 5.22T–1.45(R*T)–0.085E + 0.026N + 0.06X [R ^2^=0.97]            (2)

Where: AC is predicted ash content (%), R is rice, T is teff, E, N and X were Edeget, Nerica-4 and X-jigna rice varieties, respectively.

**Figure 1.**
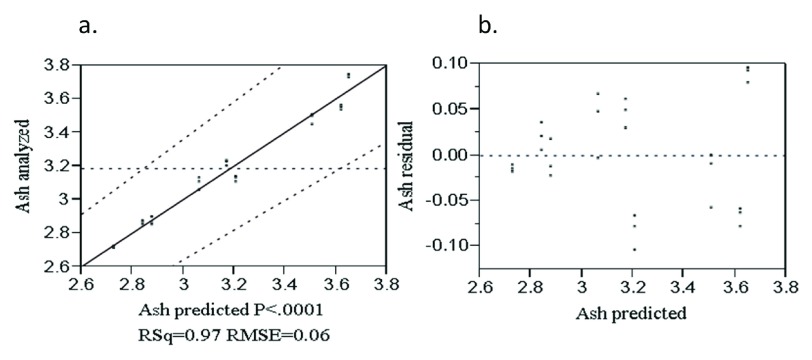
Analyzed value versus predicted ( **a**) and residual versus predicted ( **b**) plot of ash (%).

The analyzed value versus predicted plot of ash content ( Figure 1a) was randomly distributed along the diagonal line with the regression coefficient of R ^2 ^= 0.97. Figure 1b shows the values of the residuals based on the fitted model. The points were randomly distributed about the zero value line on the vertical axis which indicates the fitted model was adequate to describe the data.

The crude fiber content of the blended products ranged from 0.63 to 1.60% ( Table 1) which is higher than 100% wheat flour bread (0.29%) ( Mongi *et al.,* 2011). The crude fiber content of the control was 0.46%. Blending rice with teff significantly (P<0.05) increased the crude fiber content of the product ( Table 1). This is due to the high fiber content of teff grain. The highest value of crude fiber was obtained when 50% Edeget rice variety and 50% teff were blended. The lowest value was obtained when 90% Edeget rice variety and 10% teff were blended.

The combined effect of rice variety and blending proportion on crude fiber were significant (P<0.0001). The linear terms of rice and the interaction term (R*T) were significant (P<0.05) on crude fiber content. The effect of the linear terms of teff and rice varieties was not significant (P>0.05) on the fiber content of the product ( Appendix table 2.4). The following model ( Equation 3) was developed to predict crude fiber.

CF = 0.34R – 0.27T + 5.87(R*T) – 0.02E + 0.06N – 0.04X [R ^2 ^= 0.85]            (3)

Where: CF is crude fiber (%) predicted, R is rice, T is teff, and E, N and X are Edeget, Nerica-4 and X-jigna rice varieties, respectively.

**Figure 2.**
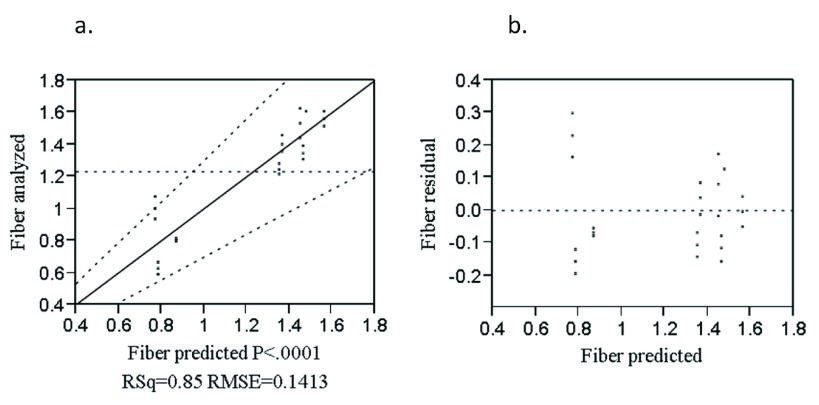
Analyzed value versus predicted ( **a**) and residual versus predicted ( **b**) plot of fiber (%).

The analyzed value versus predicted plot to crude fiber ( Figure 2a) was well modeled at regression coefficient of (R ^2 ^= 0.85). The points were randomly distributed around the diagonal line which indicates the good fits of the model to the results. The residual versus predicted plot to crude fiber is shown in Figure 2b. The points were scattered on the zero value of the horizontal line indicating that the model is adequate to describe the data.

The crude fat content of the products ranged from 0.85 to 1.90% which is higher than 30% cocoyam-wheat composite bread (0.54%) and lower than 100% wheat bread (2.02%) ( Mongi *et al.,* 2011). The crude fat content of the control (0.63%) was significantly (P<0.05) increased on blending with teff; because the crude fat content of teff is higher than rice ( Table 1). High crude fat content was obtained when 50% Edeget rice variety and 50% teff were blended and low value was obtained when 90% Edeget rice variety and 10% teff were blended.

The combined effect of the rice variety and blending proportion on crude fat content was significant (P<0.0001). The estimated parameters of linear terms and interaction (R*T) term were significant for crude fat content (P<0.0001) ( Appendix table 3.4). Rice varieties Edeget, Nerica-4 and X-jigna had no significant effect on crude fat content (P<0.05). The following model ( Equation 4) was developed to predict crude fat content of the product.

F = 0.81R + 4.41T – 3.28 (R*T) + 0.004E – 0.015N + 0.01X [R ^2 ^= 0.95]            (4)

Where: F is crude fat (%), R is rice, T is teff and E, N and X are Edeget, Nerica-4 and X-jigna rice varieties, respectively.

**Figure 3.**
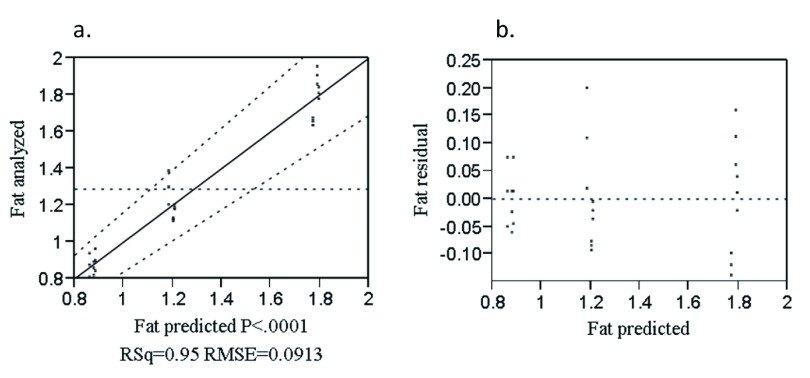
Analyzed value versus predicted ( **a**) and residual versus predicted ( **b**) plot of fat (%).

The analyzed value versus predicted plot ( Figure 3a) was randomly distributed nearby the diagonal line which indicates the goodness of fit of the model. The residual versus predicted plot ( Figure 3b) shows data points randomly distributed over the zero valued horizontal line which indicates that the model was adequate in describing the data.

The analysis shows that the protein content is significantly (P<0.05) increased as the proportion of teff increased. The protein content of the rice-teff blend bread had ranged from 9.71–10.75% ( Table 1). All blended products were found to have higher crude protein contents than the control (9.74%) except Edeget rice variety (9.71%) which was blended at 50% with 50% teff ( Table 1). This study shows that the crude protein content of rice-teff blend bread is lower than 100% wheat bread (12.54%) and higher than 30% cocoyam-wheat composite bread (9.04%) ( Mongi *et al.,* 2011).

The combined effect of the rice variety and blending proportion on crude protein was insignificant (P>0.05) ( Appendix table 4.2). The linear terms of rice and teff had a significant effect on crude protein content (P<0.0001). Inclusion of the Edeget rice variety had a significant effect on crude protein (P<0.05) content. The following model ( Equation 5) was developed to predict the crude protein content of the product.

P = 9.98R + 8.43T + 4.82(R*T) – 0.19E + 0.13N + 0.09X [R ^2^ = 0.60]            (5)

Where: P is crude protein (%), R is rice, T is teff and E, N and X are Edeget, Nerica-4 and X-jigna rice varieties, respectively.

**Figure 4.**
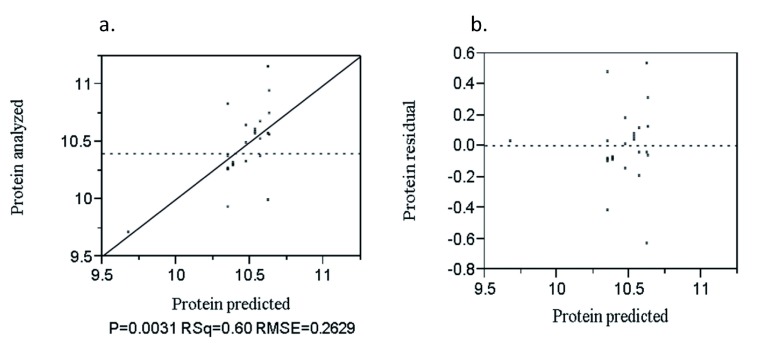
Analyzed value versus predicted ( **a**) and residual versus predicted ( **b**) plot of protein (%).

The analyzed value versus predicted plot to protein is presented in Figure 4a. The determinant coefficient (R ^2^) was 60%. The residual versus predicted plot to protein was presented in Figure 4b. The points were randomly distributed about the zero value horizontal line.

The carbohydrate content of rice-teff blend bread ranged from 77.84–81.37% ( Table 1). This is higher than the carbohydrate content of 100% wheat bread which is 63.25% ( Mongi *et al.,* 2011). A significant (P<0.05) decrease in carbohydrate content was observed with an increase in teff proportion ( Table 1). This may be due to the fact that rice flour is higher in carbohydrate as compared to teff flour ( Edeogu *et al.* 2007). The carbohydrate content of the control (X-jigna rice variety) was 81.78% which was significantly (P<0.05) decreased when blended with teff ( Table 1). The lowest carbohydrate content (77.84%) was obtained when 50% Edeget rice was blended with 50% teff. Odetokun (2000) had reported that the increase in carbohydrate content during fermentation might be due to a reduction in the fiber content and an increase in both reducing sugars and total soluble sugars. These observations may also be attributed to the fact that during fermentation carbohydrate including cellulose, pectin, lignocellulose and starch are broken down by fermenting microorganisms thereby reducing the fiber content of such food ( Raimbault & Tewe, 2001).

The combined effect of the rice variety and blending proportion on carbohydrate content was significant (P<0.0001) ( Appendix table 5.2). The linear terms of rice and teff were significant (P<0.0001). The interaction term (R*T) and Edeget rice variety did not differ significantly on carbohydrate content (P>0.05). The following model ( Equation 6) was developed to predict carbohydrate content of the product.

CHO = 81.77R + 78.39T – 7(R*T) + 0.15E + 0.31N – 0.46X [R ^2 ^= 0.84]            (6)

Where: CHO is predicted carbohydrate (%), R is rice, T is teff and E, N and X are Edeget, Nerica-4 and X-jigna rice varieties, respectively.

**Figure 5.**
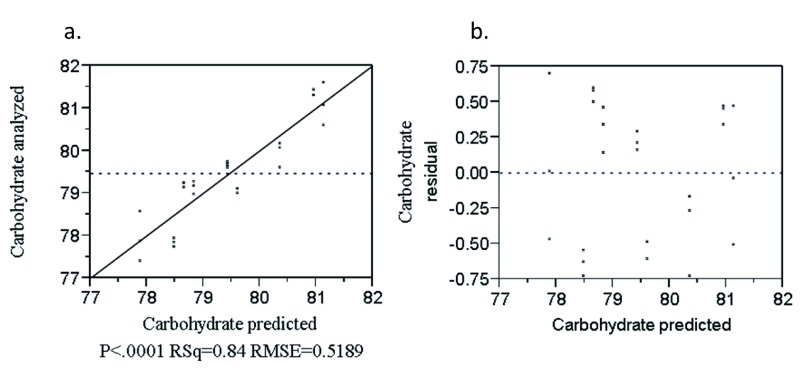
Analyzed value versus predicted ( **a**) and residual versus predicted ( **b**) plot of carbohydrate (%).

Figure 5a shows the points were randomly distributed near to the diagonal line with regression coefficient of R ^2 ^= 0.84 which indicates the goodness of fit of the model to the data. The residual by predicted plot is presented in Figure 5b. The points were randomly distributed about the zero value horizontal line which indicates that the model was adequate in describing the data.

### The effect of rice variety and blending proportion on minerals and phytic acid contents

The iron content of rice-teff blended bread is shown in Table 2. The values ranged from 2.73–12.97 mg/100g. The iron content of the product was significantly (P<0.05) increased on blending with teff. The increase in iron content is due to the high iron content of teff compared to rice ( Abebe *et al.*, 2007). A maximum value of iron was obtained when 50% Nerica-4 and 50% teff were blended and a minimum value was obtained at 90% Edeget and 10% teff. An increase in iron after fermentation may be due to a reduction of phytate during fermentation. This is because fermentation is known to reduce phytate that forms complexes with different minerals and in part contributed by high iron contents of grain teff ( Abebe *et al.*, 2007).

**Table 2.** The effect of rice variety and blending proportion on minerals and phytic acid contents.

Runs	Ingredients		V	Fe (mg/100g)	Zn (mg/100g)	Ca (mg/100g)	Phytic acid (mg/g)
	Rice	Teff					
1	0.5	0.5	E	11.22±0.56 ^c^	3.90±0.10 ^b^	61.25±0.26 ^a^	0.52±0.02 ^c^
2	0.7	0.3	E	7.96±0.05 ^d^	2.70±0.02 ^g^	46.72±0.91 ^c^	0.41±0.01 ^e^
3	0.9	0.1	E	2.73±0.11 ^h^	3.69±0.01 ^c^	26.01±0.30 ^f^	0.36±0.01 ^f^
4	0.5	0.5	X	11.75±0.17 ^b^	4.14±0.09 ^a^	59.10±0.29 ^b^	0.62±0.01 ^a^
5	0.7	0.3	X	8.04±0.22 ^d^	2.69±0.08 ^g^	47.19±0.64 ^c^	0.56±0.01 ^b^
6	0.9	0.1	X	5.70±0.18 ^e^	2.95±0.06 ^f^	28.02±0.11 ^e^	0.35±0.01 ^f^
7	0.5	0.5	N	12.97±0.15 ^a^	3.56±0.02 ^d^	59.99±0.83 ^b^	0.53±0.01 ^c^
8	0.7	0.3	N	4.77±0.00 ^f^	2.98±0.03 ^f^	44.44±0.57 ^d^	0.44±0.01 ^d^
9	0.9	0.1	N	3.12±0.09 ^g^	3.73±0.03 ^c^	25.31±0.64 ^f^	0.31±0.01 ^g^
Mean				6.83±4.16	3.38±0.50	4.60±1.59	0.43±0.12
Range				2.73–12.97	2.70–4.14	25.31–61.25	0.31–0.62
Control	1	0	X	0.00±0.00 ^i^	3.46±0.00 ^e^	17.97±0.00 ^g^	0.21±0.01 ^h^

Values are in means ± standard deviation on dry matter basis. Means within a column with the same letter are not significantly different at 95% probability levels. Where: V=rice variety, E=Edeget, X=X-jigna, N=Nerica-4, Ca=calcium, Fe=iron and Zn=zinc.

The combined effect of the rice variety and blending proportion was significant (P<0.0001) ( Appendix table 6.2). The linear terms of rice (P<0.05) and teff (P<0.0001) had a significant effect on iron content ( Appendix table 6.4). The interaction term (R*T) and X-jigna rice variety had a significant effect (P<0.05) on the iron content of the product. The inclusion of Edeget and Nerica-4 rice varieties had no significant effect (P>0.05) on the iron content of the product. The iron prediction model was developed as shown in Equation 7 below.

Fe = 4.78R + 41.9T – 45.44 (R*T) – 0.28E – 0.63N + 0.91X [R ^2 ^= 0.84]            (7)

Where: Fe is predicted iron (mg/100g), R is rice, T is teff and E, N and X are Edeget, Nerica-4 and X-jigna rice varieties, respectively.

**Figure 6.**
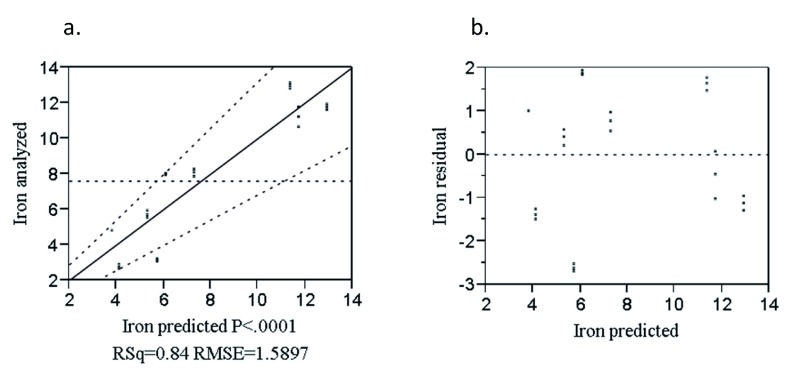
Analyzed value versus predicted ( **a**) and residual versus predicted ( **b**) plot of iron (%).

The analyzed and predicted values of iron ( Figure 6a) were closely correlated with the data as demonstrated by regression coefficient (R ^2^ = 0.84). The majority of the points were randomly distributed nearby the diagonal line which indicates the goodness of fit of the model. The residual versus predicted plots for iron ( Figure 6b) were randomly distributed about the zero value horizontal line on the vertical axis. This indicates that the model was adequate in describing the data.

Iron carries oxygen to the cells and it is necessary for the production of energy, the synthesis of collagen and the functioning of the immune system. Iron deficiency is a global problem with children and pre-menopausal women are highly affected. However, great care must also be taken not to take too much iron, as excess amounts are stored in the body’s tissues and adversely affect the body’s immune function, cell growth and heart health ( Halliday, 1998; Rebouche *et al.,* 1999).

Iron absorption can be influenced by calcium, magnesium, manganese, zinc, anti-acids and tetracycline (a common antibiotic) ( Agency of Toxic Substances and Disease Registry, 2004). Iron deficiency deprives body tissues of oxygen and results in anemia which is characterized by low blood iron level, small red blood cells and low blood hemoglobin values. Outward effects of anemia include; fatigue, paleness, dizziness, sensitivity to cold, irritability, poor concentration and heart palpitation ( Agency of Toxic Substances and Disease Registry, 2004). Recommended daily allowance of iron depending on age level and health condition is 10 to 30 mg and the recommended daily intake is 15 mg. The iron content of rice-teff blend bread was within the recommended range ( Table 2).

The zinc content of the blended products ranged from 2.70 to 4.14mg/100g ( Table 2). The analysis illustrated that there was a significant (P<0.05) difference in zinc content of the product between blends of rice and teff. The zinc content of the control was 3.46mg/100g. The highest value (4.14mg/100g) was obtained when 50% X-jigna rice variety and 50% teff were blended and the lowest value was obtained when 70% Edeget rice variety and 30% teff were blended. Fermentation has been reported to significantly increase zinc solubility (2 to 28%) and zinc uptake by intestinal segment from 1 to 16% ( Agte *et al.,* 1997). This may be due to the microbial fermentation, which enhances zinc bioavailability through hydrolysis induced by microbial phytase enzymes ( Walingo, 2009).

The combined effect of the rice variety and blending proportion was significant (P<0.0001). The linear terms of rice and teff and the interaction term (R*T) had a significant effect on zinc content (P<0.0001) ( Appendix table 7.4). The difference between rice varieties was insignificant (P>0.05) for zinc content. The model used to predict zinc content was presented by Equation 8 as follows.

Zn = 4.45R + 14.21T – 21.83(R*T) + 0.06E + 0.052N – 0.112X [R ^2 ^= 0.76]            (8)

Where: Zn is zinc (mg/100g), R is rice, T is teff and E, N and X were Edeget, Nerica-4 and X-jigna rice varieties, respectively.

**Figure 7.**
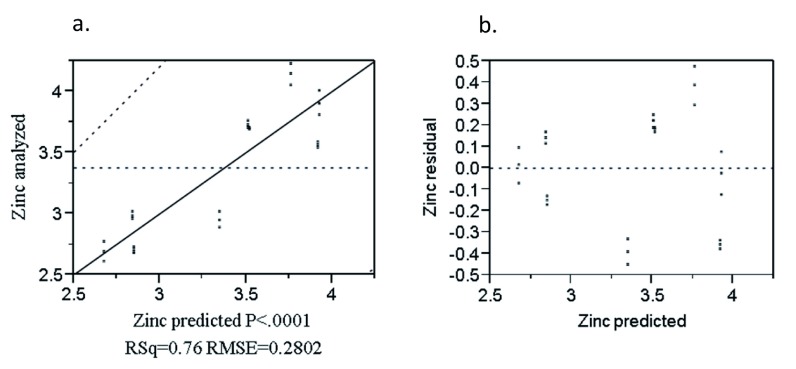
Analyzed value versus predicted ( **a**) and residual versus predicted ( **b**) plot of zinc (%).

The analyzed value versus predicted plot for zinc is shown in Figure 7a. Both values were closely correlated (R ^2 ^= 0.76). This indicates the majority of the points were randomly distributed near the diagonal line which indicates the goodness of fit of the model. The residual versus predicted plot for zinc ( Figure 7b) was randomly distributed about the zero value horizontal line on the vertical axis. This indicates that the model was adequate in describing the data.

Zinc is an essential micronutrient for animals, plants and microorganisms. Organisms can accumulate a considerable amount of zinc in their system without any damaging effect ( Agency for Toxic Substances and Disease Registry, 1995). It is essential for carbohydrate metabolism, protein synthesis and inter-nodal elongation (stem growth). Zinc participates in all major biochemical pathways and plays multiple roles in the perpetuation of genetic material, and ultimately cell division. When the supply of dietary zinc is insufficient to support these functions, biochemical abnormalities and clinical signs of zinc mal-absorption occur. Zinc deficiency leads to iron deficiency causing similar symptoms to anemia; loss of appetite, growth retardation and immunological abnormalities ( Agency of Toxic Substances and Disease Registry, 2004; Craig, 1994; Kimura & Itokawa, 1990).

The recommended daily allowance of zinc is 15 mg/day for men and 12 mg/day for women. Recent research suggests that men have a higher need for zinc than do women. Thus, it is appropriate that the recommended daily allowance is sex-specific for zinc ( Kimura & Itokawa, 1990).

The calcium content of the blended products ranged from 25.31 to 61.25mg/100g ( Table 2). The analysis indicated that means with in a column were significantly (P<0.05) different and this shows that the calcium content is significantly different between products. All the blends had higher calcium content than the control (17.97mg/100g). The highest value (61.25mg/100g) was obtained when 50% Edeget rice variety and 50% teff were blended and the lowest value was obtained when 90% Nerica-4 rice variety and 10% teff were blended. The observed high calcium content may be contributed by high calcium content of teff ( Umeta *et al.,* 2005).

The combined effect of the rice variety and blending proportion on calcium content was significant (P<0.0001) ( Appendix table 8.2). The linear terms of rice and teff and the interaction term (R*T) had a significant effect on calcium content (P<0.0001). The inclusion of the Nerica-4 rice variety had a significant effect (P<0.05) while the inclusion of Edeget and X-jigna rice varieties were insignificant (P>0.05) on the calcium content of the product. The model used to predict calcium content was presented by Equation 9 as follows.

Ca = 14.49R + 70.32T + 70.84(R*T) + 0.43E – 0.98N + 0.54X [R ^2 ^= 0.98]            (9)

Where: Ca is calcium (mg/100g), R is rice, T is teff and E, N and X are Edeget, Nerica-4 and X-jigna rice varieties, respectively.

Calcium forms a vital part of bone and tooth structure and it is important as a positive ion (Ca ^2+^) in blood clotting, muscle contraction and nerve impulse transmission. It also participates in glycogen metabolism ( Krebs-Smith *et al.,* 1997; WHO, 1996). Inadequate intake of calcium increases the risk of osteoporosis (bone loss with no apparent cause). Excess intake of calcium may cause kidney stones and reduces mineral absorption in general. The recommended dietary allowance of Calcium for adult is 800 mg; for pregnant women and young adults it is 1200 mg ( Tortora, 1997).

**Figure 8.**
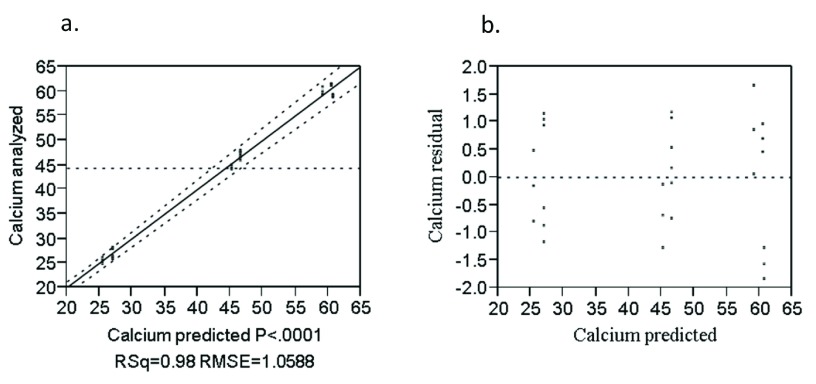
Analyzed value versus predicted ( **a**) and residual versus predicted ( **b**) plot of calcium (%).

The analyzed value versus predicted plot to calcium is given in Figure 8a. The values were strongly correlated (R ^2 ^= 0.98). The residual versus predicted plots for calcium ( Figure 8b) were randomly distributed about the zero value horizontal line on the vertical axis. This indicates that the model was adequate in describing the data.

The phytic acid content of the control and blend is presented in Table 2. There was a significant (P<0.05) differences in phytic acid content among the blended products. Values ranged from 0.31–0.62mg/g. The phytic acid content of the control was 0.21mg/g. Natural fermentation can achieve a large reduction in phytic acid by the action of bacterial as well as grain phytases. These reduce the hexa form of phytic acid into lower forms, which have a lower binding capacity for metals like iron and zinc ( Agte *et al.,* 1999). Results of fermentation on wheat bread showed that it could significantly improve *in vivo* bioavailability of minerals ( Turk *et al.,* 2000).

The decrease in phytate content could be attributed to possible secretion of the hydrolytic enzyme (phytase) by microorganisms. This enzyme is capable of hydrolyzing phytate content in the fermented foods ( Ojokoh *et al.*, 2005).

The combined effect of rice variety and blending proportion on phytic acid content was significant (P<0.0001) ( Appendix table 9.2). The linear terms of rice and X-jigna rice variety had a significant effect on phytic acid (P<0.0001). The linear term of teff, Edeget and Nerica-4 rice varieties had significant effect on phytic acid (P<0.05). Fermentation of grains significantly decreased the phytic acid content of the blended product. The most marked reduction of phytic acid in the product was obtained at proportion of 90% Nerica-4 rice variety to 10% teff. The following model was developed to predict the phytic acid content of the product as shown by Equation 10.

PA = 0.25R + 0.56T + 0.6(R*T) – 0.026E – 0.027N + 0.053X [R ^2^ = 0.92]            (10)

Where: PA is phytic acid (mg/g), R is rice, T is teff and E, N and X are Edeget, Nerica-4 and X-jigna rice variety, respectively.

**Figure 9.**
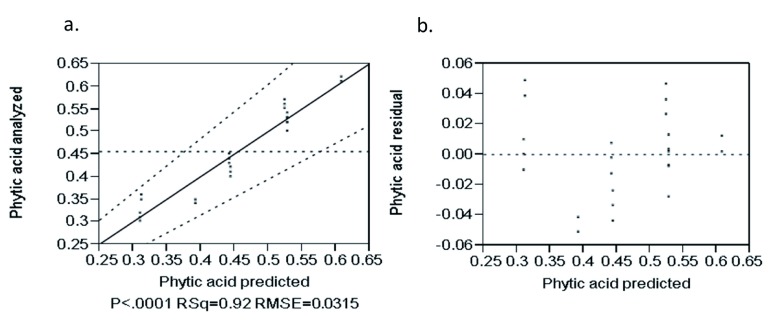
Analyzed value versus predicted ( **a**) and residual versus predicted ( **b**) plot of phytic acid (%).

The analyzed versus predicted value plots for phytic acid ( Figure 9a) were closely correlated by the regression coefficient (R ^2^ = 0.92) and the points were randomly distributed near by the diagonal line which indicates the goodness of fit of the model. The residual versus predicted value plot for phytic acid ( Figure 9b) were randomly distributed about the zero value horizontal line which indicates that the model was adequate in describing the data.

Proximate compositions, minerals, and phytic acid contents of rice-teff blend bread Nutrient, mineral and phytic acid compositions of the rice-teff blends as determined through proximate analysis. Where: N is Nerica-4 rice variety, X is X-jigna rice variety, E is Edeget rice variety, and CHO is Carbohydrate ( Legesse *et al.*, 2015c). Click here for additional data file. Copyright: © 2015 Legesse S et al. 2015 Data associated with the article are available under the terms of the Creative Commons Zero "No rights reserved" data waiver (CC0 1.0 Public domain dedication).

## Conclusions

This study revealed that rice varieties and blending proportion leads to significant difference in the proximate compositions, minerals and phytic acid contents of rice-teff blend bread. Therefore, blending of rice and teff in different proportions when making bread can compensate for the limitation of whole rice bread and whole teff bread. The combined effect of rice variety and blending proportions were significant (P<0.0001) in all the responses analyzed except protein. Carbohydrate values were significantly decreased with an increasing proportion of teff (P<0.05) for all varieties of rice, as rice has a higher carbohydrate content than teff. Ash, crude fiber and fat content significantly increased (P<0.05) with the increased proportion of teff blend. Addition of teff to rice significantly increased the iron, zinc, calcium and phytic acid contents of the product. The protein content of the bread product was not significantly influenced by the rice variety and blending proportion of rice and teff (P>0.05).

The regression coefficient (R ^2^) values of ash content, fat, calcium and phytic acid were greater than 0.90. All the derived mathematical models for the various responses were found to be fit significantly to predicted data.

## Data availability

The data referenced by this article are under copyright with the following copyright statement: Copyright: © 2015 Legesse S et al.

Data associated with the article are available under the terms of the Creative Commons Zero "No rights reserved" data waiver (CC0 1.0 Public domain dedication). 

F1000Research: Dataset 1. Data for JMP analysis, 10.5256/f1000research.6201.d45224 ( Legesse *et al.*, 2015a).

F1000Research: Dataset 2. Data for SAS analysis, 10.5256/f1000research.6201.d45225 ( Legesse *et al.*, 2015b).

F1000Research: Dataset 3. Proximate compositions, minerals, and phytic acid contents of rice-teff blend bread, 10.5256/f1000research.6201.d45226 ( Legesse *et al.*, 2015c).

## Figures and Tables

**Appendix table 1.1.** Summary of fit for ash content.

RSquare	0.972936
RSquare Adj	0.968015
Root Mean Square Error	0.059978
Mean of Response	3.18407
Observations	27

**Appendix table 1.2.** ANOVA for ash content.

Source	DF	Sum of Squares	Mean Square	F Ratio	Prob > F
Model	4	2.8451335	0.711283	197.7231	<.0001*
Error	22	0.0791422	0.003597		
C. Total	26	2.9242757			

**Appendix table 1.3.** Lack of fit for ash content.

Source	DF	Sum of Squares	Mean Square	F Ratio	Prob > F	Max RSq
Lack of Fit	4	0.07165339	0.017913	43.0564	<.0001*	0.9974
Pure Error	18	0.00748878	0.000416			
Total Error	22	0.07914217				

**Appendix table 1.4.** Parameters estimated for ash content.

Term	Estimate	Std Error	t Ratio	Prob>t
R	2.6931722	0.045672	58.97	<.0001*
T	5.2190056	0.288144	18.11	<.0001*
R*T	-1.455556	0.612149	-2.38	0.0265*
Variety[E]	-0.085848	0.016324	-5.26	<.0001*
Variety[N]	0.0255852	0.016324	1.57	0.1313
Variety[X]	0.060263	0.016324	3.69	0.0013*

**Appendix table 2.1.** Summary of fit for crude fiber content.

RSquare	0.852692
RSquare Adj	0.825909
Root Mean Square Error	0.141272
Mean of Response	1.234733
Observations	27

**Appendix table 2.2.** ANOVA for crude fiber content.

Source	DF	Sum of Squares	Mean Square	F Ratio	Prob > F
Model	4	2.5415584	0.635390	31.8369	<.0001*
Error	22	0.4390689	0.019958		
C. Total	26	2.9806274			

**Appendix table 2.3.** Lack of fit for crude fiber content.

Source	DF	Sum of Squares	Mean Square	F Ratio	Prob > F	Max RSq
Lack of Fit	4	0.39414547	0.098536	39.4817	<.0001*	0.9849
Pure Error	18	0.04492345	0.002496			
Total Error	22	0.43906892				

**Appendix table 2.4.** Parameters estimated for crude fiber content.

Term	Estimate	Std Error	t Ratio	Prob>t
R	0.3430972	0.107576	3.19	0.0042*
T	-0.275569	0.67869	-0.41	0.6886
R*T	5.8758333	1.441848	4.08	0.0005*
Variety[E]	-0.024	0.038449	-0.62	0.5389
Variety[N]	0.0610444	0.038449	1.59	0.1266
Variety[X]	-0.037044	0.038449	-0.96	0.3458

**Appendix table 3.1.** Summary of fit for crude fat content.

RSquare	0.954552
RSquare Adj	0.946288
Root Mean Square Error	0.091321
Mean of Response	1.287393
Observations	27

**Appendix table 3.2.** ANOVA for crude fat content.

Source	DF	Sum of Squares	Mean Square	F Ratio	Prob > F
Model	4	3.8534586	0.963365	115.5167	<.0001*
Error	22	0.1834716	0.008340		
C. Total	26	4.0369302			

**Appendix table 3.3.** Lack of fit for crude fat content.

Source	DF	Sum of Squares	Mean Square	F Ratio	Prob > F	Max RSq
Lack of Fit	4	0.14091525	0.035229	14.9007	<.0001*	0.9895
Pure Error	18	0.04255630	0.002364			
Total Error	22	0.18347155				

**Appendix table 3.4.** Parameters estimated for crude fat content.

Term	Estimate	Std Error	t Ratio	Prob>t
R	0.8111068	0.06954	11.66	<.0001*
T	4.4064297	0.438722	10.04	<.0001*
R*T	-3.28533	0.932046	-3.52	0.0019*
Variety[E]	0.0044189	0.024855	0.18	0.8605
Variety[N]	-0.014724	0.024855	-0.59	0.5596
Variety[X]	0.0103054	0.024855	0.41	0.6824

**Appendix table 4.1.** Summary of fit for crude protein content.

RSquare	0.595766
RSquare Adj	0.474496
Root Mean Square Error	0.262899
Mean of Response	10.39862
Observations	27

**Appendix table 4.2.** ANOVA for crude protein content.

Source	DF	Sum of Squares	Mean Square	F Ratio	Prob > F
Model	4	0.8758976	0.218974	1.8939	0.1473
Error	22	2.5437081	0.115623		
C. Total	26	3.4196057			

**Appendix table 4.3.** Lack of fit for crude protein content.

Source	DF	Sum of Squares	Mean Square	F Ratio	Prob > F	Max RSq
Lack of Fit	4	1.2810090	0.320252	4.5653	0.0101*	0.6307
Pure Error	18	1.2626992	0.070150			
Total Error	22	2.5437081				

**Appendix table 4.4.** Parameters estimated for crude protein content.

Term	Estimate	Std Error	t Ratio	Prob>t
R	9.9791492	0.258931	38.54	<.0001*
T	8.4334645	1.633574	5.16	<.0001*
R*T	4.8173376	3.470457	1.39	0.1790
Variety[E]	-0.197796	0.092546	-2.14	0.0439*
Variety[N]	0.1324749	0.092546	1.43	0.1664
Variety[X]	0.0653216	0.092546	0.71	0.4877

**Appendix table 5.1.** Summary of fit for carbohydrate.

RSquare	0.839604
RSquare Adj	0.810442
Root Mean Square Error	0.518943
Mean of Response	79.47388
Observations	27

**Appendix table 5.2.** ANOVA for carbohydrate.

Source	DF	Sum of Squares	Mean Square	F Ratio	Prob > F
Model	4	31.013066	7.75327	28.7902	<.0001*
Error	22	5.924647	0.26930		
C. Total	26	36.937713			

**Appendix table 5.3.** Lack of fit for carbohydrate.

Source	DF	Sum of Squares	Mean Square	F Ratio	Prob > F	Max RSq
Lack of Fit	4	4.4659416	1.11649	13.7771	<.0001*	0.9605
Pure Error	18	1.4587057	0.08104			
Total Error	22	5.9246473				

**Appendix table 5.4.** Parameters estimated for carbohydrate.

Term	Estimate	Std Error	t Ratio	Prob>t
R	81.772845	0.395168	206.93	<.0001*
T	78.393291	2.493082	31.44	<.0001*
R*T	-7.009647	5.296443	-1.32	0.1993
Variety[E]	0.1496183	0.141238	1.06	0.3009
Variety[N]	0.3132235	0.141238	2.22	0.0372*
Variety[X]	-0.462842	0.141238	-3.28	0.0034*

**Appendix table 6.1.** Summary of fit for iron content.

RSquare	0.839085
RSquare Adj	0.809828
Root Mean Square Error	1.589741
Mean of Response	7.585222
Observations	27

**Appendix table 6.2:** ANOVA for iron content.

Source	DF	Sum of Squares	Mean Square	F Ratio	Prob > F
Model	4	289.92563	72.4814	28.6796	<.0001*
Error	22	55.60011	2.5273		
C. Total	26	345.52574			

**Appendix table 6.3.** Lack of fit for iron content.

Source	DF	Sum of Squares	Mean Square	F Ratio	Prob > F	Max RSq
Lack of Fit	4	54.654548	13.6636	260.1038	<.0001*	0.9973
Pure Error	18	0.945567	0.0525			
Total Error	22	55.600115				

**Appendix table 6.4.** Parameters estimated for iron content.

Term	Estimate	Std Error	t Ratio	Prob>t
R	4.7810917	1.210566	3.95	0.0007*
T	41.897592	7.637357	5.49	<.0001*
R*T	-45.44083	16.22523	-2.80	0.0104*
Variety[E]	-0.280972	0.432673	-0.65	0.5228
Variety[N]	-0.632372	0.432673	-1.46	0.1580
Variety[X]	0.9133444	0.432673	2.11	0.0464*

**Appendix table 7.1.** Summary of fit for zinc content.

RSquare	0.761491
RSquare Adj	0.718126
Root Mean Square Error	0.280153
Mean of Response	3.371689
Observations	27

**Appendix table 7.2.** ANOVA for zinc content.

Source	DF	Sum of Squares	Mean Square	F Ratio	Prob > F
Model	4	5.5128057	1.37820	17.5599	<.0001*
Error	22	1.7266813	0.07849		
C. Total	26	7.2394870			

**Appendix table 7.3.** Lack of fit for zinc content.

Source	DF	Sum of Squares	Mean Square	F Ratio	Prob > F	Max RSq
Lack of Fit	4	1.6644809	0.416120	120.4199	<.0001*	0.9914
Pure Error	18	0.0622004	0.003456			
Total Error	22	1.7266813				

**Appendix table 7.4.** Parameter estimates for zinc content.

Term	Estimate	Std Error	t Ratio	Prob>t
R	4.4456833	0.213332	20.84	<.0001*
T	14.21135	1.345896	10.56	<.0001*
R*T	-21.83833	2.859296	-7.64	<.0001*
Variety[E]	0.0600611	0.076248	0.79	0.4393
Variety[N]	0.0520944	0.076248	0.68	0.5016
Variety[X]	-0.112156	0.076248	-1.47	0.1555

**Appendix table 8.1.** Summary of fit for calcium.

RSquare	0.9775245
RSquare Adj	0.977438
Root Mean Square Error	1.058755
Mean of Response	44.22424
Observations	27

**Appendix table 8.2.** ANOVA for calcium.

Source	DF	Sum of Squares	Mean Square	F Ratio	Prob > F
Model	4	5161.6687	1290.42	1151.168	<.0001*
Error	22	24.6612	1.12		
C. Total	26	5186.3298			

**Appendix table 8.3.** Lack of fit for calcium.

Source	DF	Sum of Squares	Mean Square	F Ratio	Prob > F	Max RSq
Lack of Fit	4	18.912201	4.72805	14.8035	<.0001*	0.9989
Pure Error	18	5.748988	0.31939			
Total Error	22	24.661189				

**Appendix table 8.4.** Parameters estimated for calcium.

Term	Estimate	Std Error	t Ratio	Prob>t
R	14.487627	0.806228	17.97	<.0001*
T	70.317919	5.086421	13.82	<.0001*
R*T	70.841042	10.80588	6.56	<.0001*
Variety[E]	0.4341611	0.288157	1.51	0.1461
Variety[N]	-0.978722	0.288157	-3.40	0.0026*
Variety[X]	0.5445611	0.288157	1.89	0.0720

**Appendix table 9.1.** Summary of fit for phytic acid.

RSquare	0.920723
RSquare Adj	0.906309
Root Mean Square Error	0.031505
Mean of Response	0.454074
Observations	27

**Appendix table 9.2.** ANOVA for phytic acid.

Source	DF	Sum of Squares	Mean Square	F Ratio	Prob > F
Model	4	0.25361481	0.063404	63.8769	<.0001*
Error	22	0.02183704	0.000993		
C. Total	26	0.27545185			

**Appendix table 9.3.** Lack of fit for phytic acid.

Source	DF	Sum of Squares	Mean Square	F Ratio	Prob > F	Max RSq
Lack of Fit	4	0.02017037	0.005043	54.4600	<.0001*	0.9939
Pure Error	18	0.00166667	0.000093			
Total Error	22	0.02183704				

**Appendix table 9.4.** Parameters estimated for phytic acid.

Term	Estimate	Std Error	t Ratio	Prob>t
R	0.25375	0.023991	10.58	<.0001*
T	0.5565278	0.151357	3.68	0.0013*
R*T	0.5972222	0.321551	1.86	0.0767
Variety[E]	-0.026296	0.008575	-3.07	0.0056*
Variety[N]	-0.027407	0.008575	-3.20	0.0042*
Variety[X]	0.0537037	0.008575	6.26	<.0001*

## Appendix

**Least squares fit to proximate compositions, minerals and phytic acid contents**

**Appendix table 1. Model fitting for ash content.**

**Appendix table 2. Model fitting for crude fiber content.**

**Appendix table 3. Model fitting for crude fat content.**

**Appendix table 4. Model fitting for crude protein content.**

**Appendix table 5. Model fitting for carbohydrate.**

**Appendix table 6. Model fitting for iron content.**

**Appendix table 7. Model fitting for zinc content.**

**Appendix table 8. Model fitting for calcium.**

**Appendix table 9. Model fitting for phytic acid.**
