# Supplementary material for: Effect of rice variety and blending proportion on the proximate compositions, minerals and phytic acid contents of bread from rice-teff blend
Source: F1000Res. 2015 May 13;4:116. [Version 1] doi: 10.12688/f1000research.6201.1 (PMC6758841; doi:10.12688/f1000research.6201.1)
Supplement: Peer review history

# Peer review: Reviewer response for version 1

**DOI:** 10.5256/f1000research.6650.r53369

**Published:** 2019-09-23

**Authors:** Joseph Adubofuor

**Affiliations:** 1 Department of Food Science and Technology, Kwame Nkrumah University of Science and Technology, Kumasi, Ghana

The presentation of the research work is satisfactory with citations of the literature. Authors should consider citing some recent references starting from 2010 onwards. The study design is technically sound. The design of experiment used is appropriate in establishing the model for the variables involved. The methods used in data collection of proximate composition, mineral and phytic acid contents were outlined and explained in detail for replication by others. The statistical analysis was done appropriately to establish the interaction effect of the main factors and generate prediction equations for the responses. All the data have been provided in tables and figures. Superscripts have been indicated to show the significant differences among the samples. The conclusions drawn have been summarized clearly and are adequately supported by the results.

 The following should be addressed:

1. **Discussion on crude fat:** *The sentence*: “Rice varieties Edeget, Nerica-4 and X-jigna had no signifcant effect on crude fat content (P<0.05)”. *Comment*: the less than sign should be changed to greater than (>).
2. **Discussion on protein content:** *The sentence*: "The analysis shows that the protein content is signifcantly (P<0.05) increased as the proportion of teff increased”. *Comment*: The sentence should be changed because from Table 1 the protein content increased and decreased as the proportion of teff increased. An appropriate reason should be given to explain this trend.
3. **Discussion on Phytic acid**: *The sentence*: "There was a signifcant (P<0.05) differences in phytic acid content among the blended products” *Comment 1*: The sentence should be changed to read as follows: "There were significant differences (p<0.05) in phytic acid content among the blended products".
*Comment 2*: In all three rice varieties, decrease in teff content of the blends resulted in decrease in phytic acid content. The reason(s) which could account for this trend should be explained.

I confirm that I have read this submission and believe that I have an appropriate level of expertise to confirm that it is of an acceptable scientific standard.

# Peer review: Reviewer response for version 1

**DOI:** 10.5256/f1000research.6650.r43046

**Published:** 2019-02-12

**Authors:** Afifa Jahan

**Affiliations:** 1 Department of Food & Nutrition, Professor Jayashankar Telangana State Agricultural University, Hyderabad, Telangana, India

The research is clearly and accurately presented and the current literature is appropriately cited. The study design is appropriate and the work is technically sound. It has sufficient details of methods and analysis provided to allow replication by others. The statistical analysis and its interpretation used in the study is appropriate. The rationale for developing the products of rice usually consumed in Ethiopia is clearly explained and description of the method is clearly presented by the author. The conclusions drawn adequately support the results.

 The research is good and the paper is very elaborate. The results are confined to Ethiopia and the products developed are commonly consumed there. Hence this paper can be accepted for indexing, provided the content is modified a little and tables can be made into a single format as too much content cannot be read by any researcher. The length of paper can also be reduced. All the mineral content and analysis can be explained under one sub head. The acceptance of product developed can be mentioned in the methodology as it was replaced with rice ( *oryza sativa*). The statistical analysis is appropriate and the content is very interesting too. Overall, a positive feedback from my side.

I confirm that I have read this submission and believe that I have an appropriate level of expertise to confirm that it is of an acceptable scientific standard.
